# Exogenous 2,4-Epibrassinolide Alleviates Alkaline Stress in Cucumber by Modulating Photosynthetic Performance

**DOI:** 10.3390/plants14010054

**Published:** 2024-12-27

**Authors:** Wenjing Nie, Qinghai He, Jinzhao Ma, Hongen Guo, Qinghua Shi

**Affiliations:** 1 Shandong Engineering Research Center of Functional Crop Germplasm Innovation and Cultivation Utilization, Yantai Engineering Research Center of Plant Stem Cell Targeted Breeding, Shandong Institute of Sericulture, Yantai 264001, China; 2 Shandong Academy of Agricultural Machinery Science, Jinan 250100, China; 3 Stage Key Laboratory of Crop Biology, College of Horticulture Science and Engineering, Shandong Agricultural University, Tai’an 271018, China

**Keywords:** cucumber, alkaline stress, 2,4-epibrassinolide, photosynthesis, Calvin cycle

## Abstract

Brassinosteroids (BRs) are recognized for their ability to enhance plant salt tolerance. While considerable research has focused on their effects under neutral salt conditions, the mechanisms through which BRs regulate photosynthesis under alkaline salt stress are less well understood. This study investigates these mechanisms, examining plant growth, photosynthetic electron transport, gas exchange parameters, Calvin cycle dynamics, and the expression of key antioxidant and Calvin cycle genes under alkaline stress conditions induced by NaHCO_3_. The findings indicate that NaHCO_3_ stress substantially impairs cucumber growth and photosynthesis, significantly reducing chlorophyll content, net photosynthetic rate (Pn), stomatal conductance (Gs), transpiration rate (E), maximum photochemical efficiency (Fv/Fm), actual photochemical efficiency (ΦPSII), antenna conversion efficiency (Fv′/Fm′), and photochemical quenching coefficient (qP). This disruption suggests a severe dysregulation of the photosynthetic electron transport system, impairing electron transfer from photosystem II (PSII) to photosystem I (PSI) and subsequently the Calvin cycle. Application of exogenous 24-epibrassinolide (EBR) alleviated these effects, reducing leaf chlorosis and growth inhibition and significantly enhancing the expression of key genes within the antioxidant system (AsA-GSH cycle) and the Calvin cycle. This intervention also led to a reduction in reactive oxygen species (ROS) accumulation and improved photosynthetic performance, as evidenced by enhancements in Pn, Gs, E, Fv/Fm, ΦPSII, Fv′/Fm′, and qP. Moreover, NaHCO_3_ stress hindered chlorophyll synthesis, primarily by blocking the conversion from porphobilinogen (PBG) to uroporphyrinogen III (UroIII) and by increasing chlorophyllase (Chlase) and decreasing porphobilinogen deaminase (PBGD) activity. Exogenous EBR countered these effects by enhancing PBGD activity and reducing Chlase activity, thereby increasing chlorophyll content under stress conditions. In summary, EBR markedly mitigated the adverse effects of alkaline stress on cucumber leaf photosynthesis by stabilizing the photosynthetic electron transport system, accelerating photosynthetic electron transport, and promoting the Calvin cycle. This study provides valuable insights into the regulatory roles of BRs in enhancing plant resilience to alkaline stress.

## 1. Introduction

Photosynthesis stands as the fundamental cornerstone of plant growth and development, driving the conversion of solar energy into chemical energy [1]. This energy conversion fuels the biosynthetic pathways that lead to biomass accumulation. The intricate process includes both the light-dependent reactions and the Calvin cycle, orchestrating the carbon assimilation from CO_2_ into carbohydrates, which are essential for plant sustenance and productivity [2]. However, environmental stresses such as salt stress, drought, and extreme temperatures pose significant threats to photosynthetic efficiency [3,4]. These stressors initiate a cascade of physiological and biochemical disruptions, including stomatal closure, impaired electron transport, and oxidative damage, which collectively compromise the photosynthetic machinery [4,5]. The alteration in photosynthetic parameters under stress conditions, such as reduced chlorophyll content and disrupted photosystem activity, ultimately curtails plant growth and crop yield [6,7]. Thus, understanding the impact of environmental stress on photosynthesis is pivotal for developing strategies to enhance plant resilience and ensure food security in the face of climatic challenges.

Salt and alkaline stress significantly impairs plant physiological processes, especially photosynthesis, which is critical for growth and productivity. This stress leads to ionic imbalances and hyperosmotic stress, disrupting cellular homeostasis and nutrient assimilation, thereby reducing plant viability [8,9,10]. Elevated concentrations of Na^+^, typical of salt conditions, and HCO_3_^−^ and CO_3_^2−^ under alkaline conditions result in cellular toxicity that manifests as chlorosis and necrosis, further impairing the photosynthetic apparatus [11]. Alkaline stress, characterized by elevated pH levels, poses a more severe threat than neutral salt stress. It not only exacerbates the toxic effects by inhibiting key enzymatic activities crucial for photosynthesis but also alters the structural integrity of chloroplasts, severely diminishing photosynthetic capacity [12]. These alkaline conditions, with higher pH, precipitate essential nutrients such as calcium and magnesium, further disrupting nutrient uptake and leading to more pronounced damage compared to neutral salt stress [13].

Salt and alkaline conditions also reduce water uptake due to osmotic stress, decreasing cell turgor and inducing stomatal closure, which limits CO_2_ availability for the Calvin cycle and further diminishes photosynthetic capacity [14,15]. Additionally, oxidative stress from salt and alkaline conditions leads to an overproduction of ROS, damaging cellular structures and overwhelming plant antioxidant defenses, thus exacerbating reductions in photosynthetic efficiency [16,17,18]. Understanding the mechanisms by which plants respond to salt and alkaline stresses is crucial for developing crops with improved resilience, which is vital for maintaining agricultural productivity in affected regions.

Brassinosteroids (BRs), first discovered in the 1970s from rape pollen, are “the sixth major plant hormone”. These compounds play a pivotal role in enhancing plant growth and resilience to environmental stresses, including salinity and alkalinity [19,20]. Their impact on plant biology extends to significant improvements in photosynthetic efficiency under stress conditions, making them an essential hormone in the adaptation of plants to challenging environments.

The role of BRs in photosynthesis, particularly under stress, is mediated through their regulation of cellular processes and gene expression that are directly linked to photosynthetic machinery [21,22,23,24]. BRs enhance the stability and functionality of PSII, crucial for maintaining optimal photosynthesis during salt and alkaline stress. This includes regulating genes that encode for proteins involved in the Calvin cycle and protecting these proteins from degradation induced by stress [25,26]. At the molecular level, BRs interact with specific receptors like BRI1 (Brassinosteroid Insensitive 1), initiating signaling cascades that ultimately lead to the activation of transcription factors. These transcription factors upregulate genes responsible for stress responses, thereby enhancing cellular homeostasis and the plant’s ability to optimize nutrient and water uptake under stressful conditions [27,28,29]. Through these mechanisms, BRs not only support basic plant development but also empower plants to withstand adverse conditions by bolstering the photosynthetic apparatus. BRs promoting growth and enhancing stress tolerance through improved photosynthesis underscores their potential in agricultural facing variable environmental stresses.

Exploring the effects of BRs on photosynthesis under salt and alkaline stress conditions is critical for enhancing plant resilience and agricultural productivity [30,31,32]. While there has been significant advancement in understanding the role of BRs in neutral salt stress mitigation, the specific photosynthetic responses to alkaline stress remain less defined, particularly concerning the complex mechanisms of photosynthesis that are affected [31,33,34,35]. This study aims to bridge this gap by investigating the impact of exogenous 24-epibrassinolide (EBR) application on the photosynthetic efficiency and stress tolerance of cucumber plants subjected to alkaline stress. It focuses on elucidating the physiological and molecular mechanisms underlying these effects. The findings from this research could contribute to the development of effective strategies for enhancing the resilience and productivity of crops grown in salt- and alkaline-affected soils, thus promoting sustainable agricultural practices in regions increasingly challenged by soil salinization [22].

## 2. Results

### 2.1. Effects of EBR on the Growth of Cucumber Under NaHCO_3_ Stress

**Figure 1:**
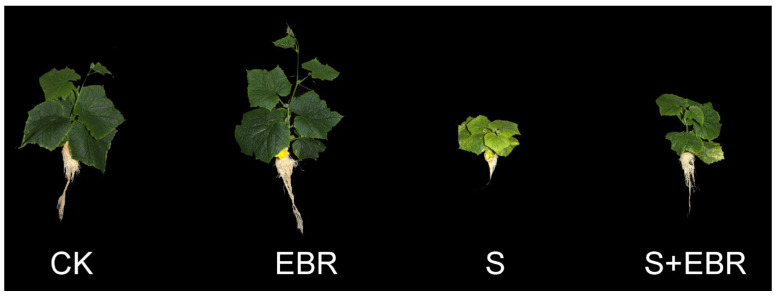
Effects of EBR on plant phenotypes under NaHCO_3_ stress. (CK denotes plants grown under normal Hoagland nutrient solution; CK + EBR denotes the addition of EBR under normal growth conditions; S denotes NaHCO_3_ stress treatment; S + EBR denotes the addition of EBR under NaHCO_3_ stress).

Exposure to NaHCO_3_ significantly inhibited the growth of cucumber seedlings, as evidenced by the yellowing and wilting of leaves (Figure 1). This treatment also resulted in a reduction in the height and both above-ground and below-ground biomass of the cucumber seedlings (*p* < 0.05) (Table 1). The application of 0.2 µM exogenous EBR markedly mitigated these inhibitory effects under NaHCO_3_ stress, enhancing the biomass of the cucumber seedlings (Table 1).

### 2.2. Effects of EBR on Reactive Oxygen Species Accumulation and Antioxidant Gene Expression in Cucumber Leaves Under NaHCO_3_ Stress

**Figure 2:**
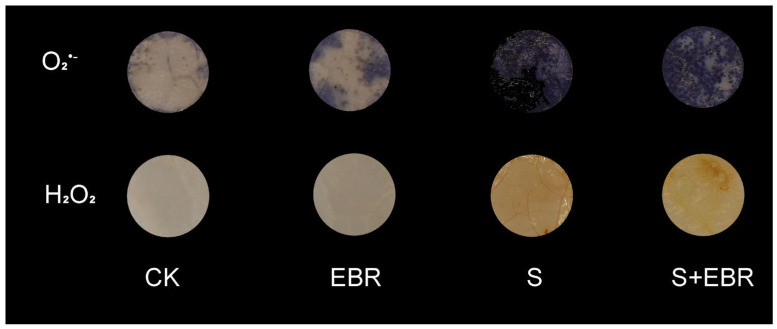
Effects of EBR on superoxide anion (O_2_^−^) and hydrogen peroxide (H_2_O_2_) staining in cucumber leaves under NaHCO_3_ stress. (CK represents control plants grown in normal Hoagland nutrient solution; EBR denotes normal growth conditions with the addition of EBR; S indicates NaHCO_3_ stress treatment; S + EBR indicates the addition of EBR under NaHCO_3_ stress). Histochemical observations utilized Nitroblue Tetrazolium (NBT) for staining superoxide anion and Diaminobenzidine (DAB) for hydrogen peroxide.

As shown in Figure 2, histochemical staining of cucumber leaves with Nitroblue Tetrazolium (NBT) for O_2_^.−^ and Diaminobenzidine (DAB) for H_2_O_2_ reveals that NaHCO_3_ stress significantly elevated the levels of both reactive oxygen species. The darker staining indicates higher concentrations of these compounds. Conversely, the application of exogenous EBR markedly reduced their accumulation under NaHCO_3_ stress, evidenced by lighter staining which corresponds to lower concentrations. These observations suggest that EBR treatment effectively mitigates oxidative damage in cucumber seedlings induced by NaHCO_3_ stress.

**Figure 3:**
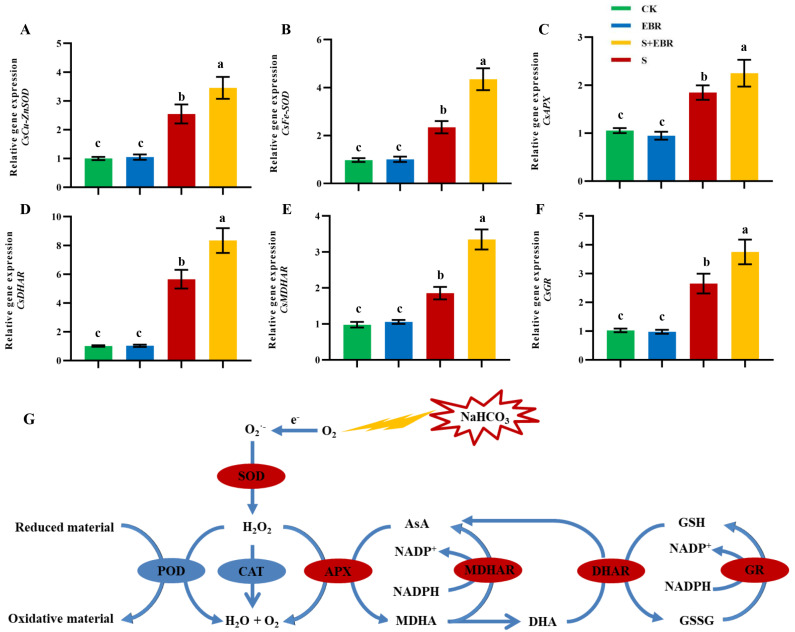
Influence of exogenous EBR on the transcript levels of key genes involved in the antioxidant and ascorbate–glutathione (ASA-GSH) cycles under NaHCO_3_ stress in cucumber leaves. Panels represent transcript levels of (**A**) *CsCu-ZnSOD*, (**B**) *CsFeSOD*, (**C**) *CsAPX*, (**D**) *CsDHAR*, (**E**) *CsMDHAR*, (**F**) *CsGR*overall, (**G**) EBR modulation of the ASA-GSH cycle. Red symbols denote upregulated genes under stress conditions. Different lowercase letters indicate statistically significant differences among treatments at the 0.05 level (*p* < 0.05, n = 3). Key abbreviations: SOD, superoxide dismutase; APX, ascorbate peroxidase; MDHAR, monodehydroascorbate reductase; DHAR, dehydroascorbate reductase; GR, glutathione reductase; AsA, ascorbic acid; MDHA, monodehydroascorbate; DHA, dehydroascorbic acid; GSH, glutathione; GSSG, oxidized glutathione. (CK represents control plants grown in normal Hoagland nutrient solution; CK + EBR denotes normal growth conditions with the addition of EBR; S indicates NaHCO_3_ stress treatment; S + EBR indicates the addition of EBR under NaHCO_3_ stress).

To elucidate the effects of EBR on the expression of gene coding for antioxidant enzymes within the AsA-GSH cycle, qRT-PCR analysis was performed. As illustrated in Figure 3, transcript levels of *CsCu-ZnSOD*, *CsFeSOD*, *CsAPX*, *CsMDHAR*, *CsDHAR*, and *CsGR* were significantly enhanced by 48 h NaHCO_3_ treatment, relative to the control. The application of EBR further upregulated the expression of these genes under NaHCO_3_ treatment. These results confirm that EBR amplifies the expression of key genes responsible for scavenging reactive oxygen species and strengthening the AsA-GSH system.

### 2.3. Effects of EBR on Photosynthetic Parameters and Chlorophyll Synthesis in Cucumber Leaves Under NaHCO_3_ Stress

**Figure 4:**
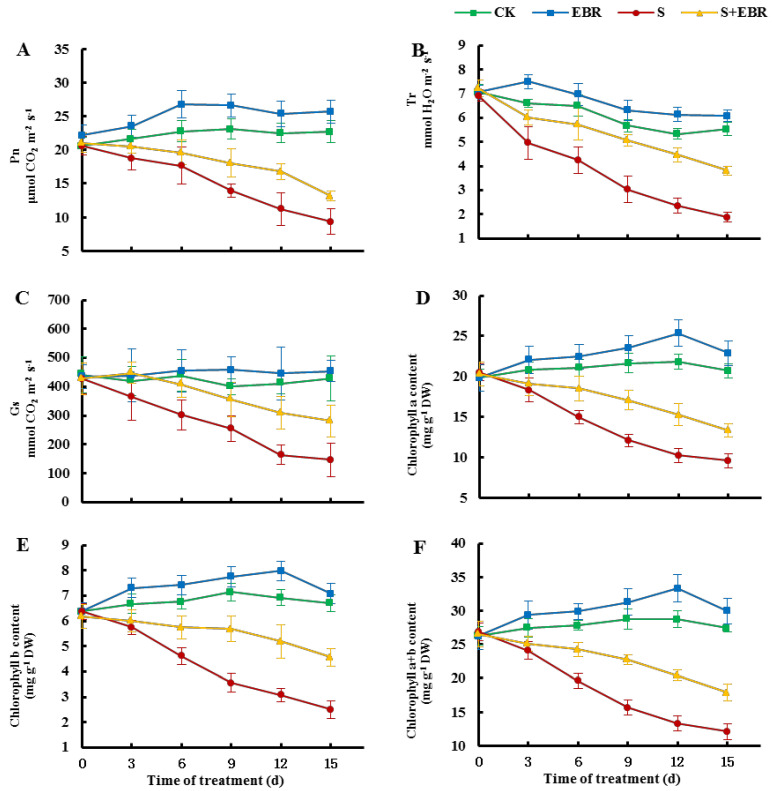
Effects of EBR on net photosynthetic rate, Pn (**A**), transpiration rate, Tr (**B**), stomatal conductance, Cs (**C**), chlorophyll a content (**D**), chlorophyll b content (**E**), and chlorophyll a + b content (**F**) of cucumber seedlings under NaHCO_3_ stress. (CK represents normal Hoagland nutrient solution culture; EBR represents the addition of EBR under normal growth conditions; S represents NaHCO_3_ stress treatment; S + EBR represents the addition of EBR under NaHCO_3_ stress).

Figure 4 shows that NaHCO_3_ stress significantly reduces photosynthetic parameters in cucumber seedling leaves, including net photosynthetic rate (Pn), transpiration rate (Tr), stomatal conductance (Gs), and chlorophyll a, chlorophyll b, and total chlorophyll contents. The application of exogenous EBR improved these indices and alleviated the inhibition of photosynthesis in cucumber seedlings under NaHCO_3_ stress, also enhancing the contents of chlorophyll a and b in the leaves.

**Figure 5:**
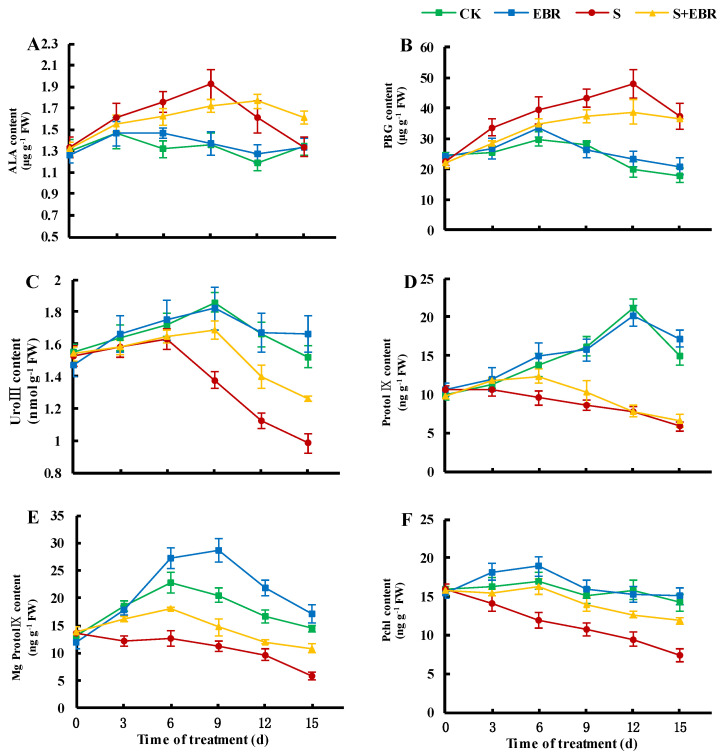
Effects of EBR on the contents of ALA (**A**), PBG (**B**), UroIII (**C**), ProtoIX (**D**), Mg-ProtoIX (**E**), and Pchl (**F**) in cucumber leaves under NaHCO_3_ stress. (CK represents normal Hoagland nutrient solution culture; EBR represents the addition of EBR under normal growth conditions; S represents NaHCO_3_ stress treatment; S + EBR represents the addition of EBR under NaHCO_3_ stress).

As depicted in Figure 5, NaHCO_3_ stress led to an increase in the contents of 5-aminolevulinic acid (ALA) and porphobilinogen (PBG), while the levels of uroporphyrinogen (UroIII), protoporphyrin (ProtoIX), magnesium protoporphyrin (Mg-ProtoIX), and protochlorophyllin acid ester (Pchl) decreased as the stress duration extended. This indicates that NaHCO_3_ stress inhibited the synthesis pathway of chlorophyll, particularly at the UroIII synthesis stage from PBG. The application of exogenous EBR resulted in increased levels of chlorophyll precursors such as UroIII, ProtoIX, Mg-ProtoIX, and Pchl and reduced the accumulation of ALA and PBG under NaHCO_3_ stress, thereby relieving the inhibition of chlorophyll synthesis and boosting chlorophyll content (Figure 4D–F).

**Figure 6:**
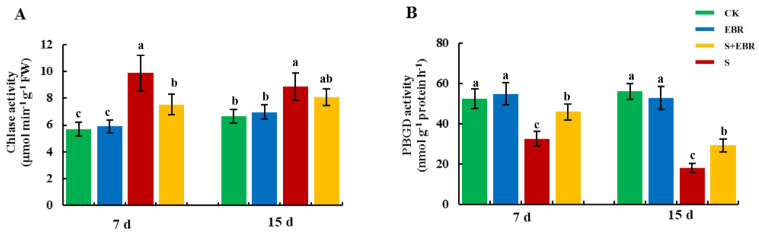
Effects of EBR on the activities of Chlase (**A**) and PBGD (**B**) in cucumber leaves under NaHCO_3_ stress. Different lowercase letters indicate statistically significant differences among treatments at the 0.05 level (*p* < 0.05, n = 3). (CK represents normal Hoagland nutrient solution culture; EBR represents the addition of EBR under normal growth conditions; S represents NaHCO_3_ stress treatment; S + EBR represents the addition of EBR under NaHCO_3_ stress).

Figure 6 illustrates that NaHCO_3_ stress significantly enhanced chlorophyllase (Chlase) activity, which could be a major factor in the reduction in chlorophyll content under alkaline stress. It also notably inhibited porphobilinogen deaminase (PBGD) activity, blocking the conversion of PBG to UROIII. Exogenous EBR application alleviated the inhibition of Chlase activity by NaHCO_3_ stress and increased PBGD activity under stress conditions.

### 2.4. Effects of EBR on the Chlorophyll Fluorescence Characteristics of Cucumber Leaves Under NaHCO_3_ Stress

**Figure 7:**
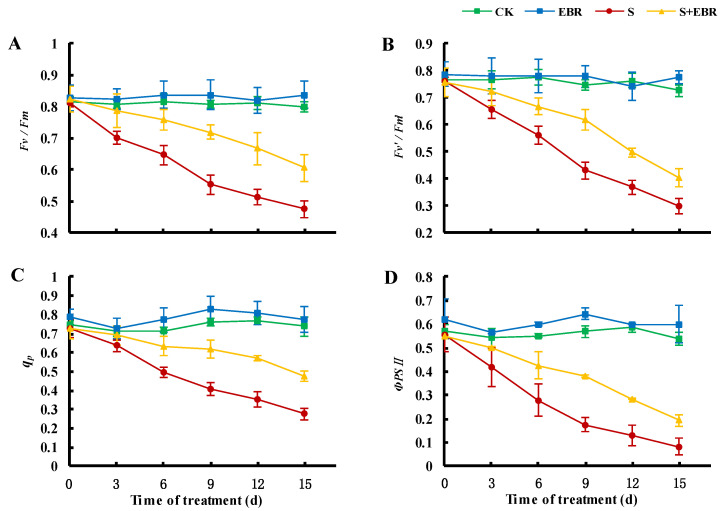
Effects of EBR on maximal photochemical efficiency of PSII, Fv/Fm (**A**); photochemical efficiency of PSII in the light, Fv′/Fm′ (**B**); photochemical quenching, qP (**C**); actual photochemical efficiency of PSII in the light, ΦPSII (**D**) in cucumber seedlings under NaHCO_3_ stress. (CK represents normal Hoagland nutrient solution culture; EBR represents the addition of EBR under normal growth conditions; S represents NaHCO_3_ stress treatment; S + EBR represents the addition of EBR under NaHCO_3_ stress).

To further investigate the regulatory mechanisms of brassinosteroids (BRs) on photosynthesis, we analyzed chlorophyll fluorescence to assess the functional state of photosystem II (PSII). The results, presented in Figure 7, show a time-dependent decrease in key photosynthetic parameters under NaHCO_3_ stress: maximum photochemical efficiency (Fv/Fm), antenna conversion efficiency (Fv′/Fm′), photochemical quenching coefficient (qP), and actual photochemical efficiency (ΦPSII) of cucumber leaves. The application of exogenous EBR significantly mitigated the stress-induced decline in these indices, suggesting a disruption in the photochemical system of PSII under NaHCO_3_ stress that impairs the absorption and utilization of light energy. Furthermore, exogenous EBR was observed to partially restore the physiological functions of the PSII photochemical system under these stress conditions.

### 2.5. Effects of EBR on the Expression of Photosynthesis-Related Genes in Cucumber Leaves Under NaHCO_3_ Stress

**Figure 8:**
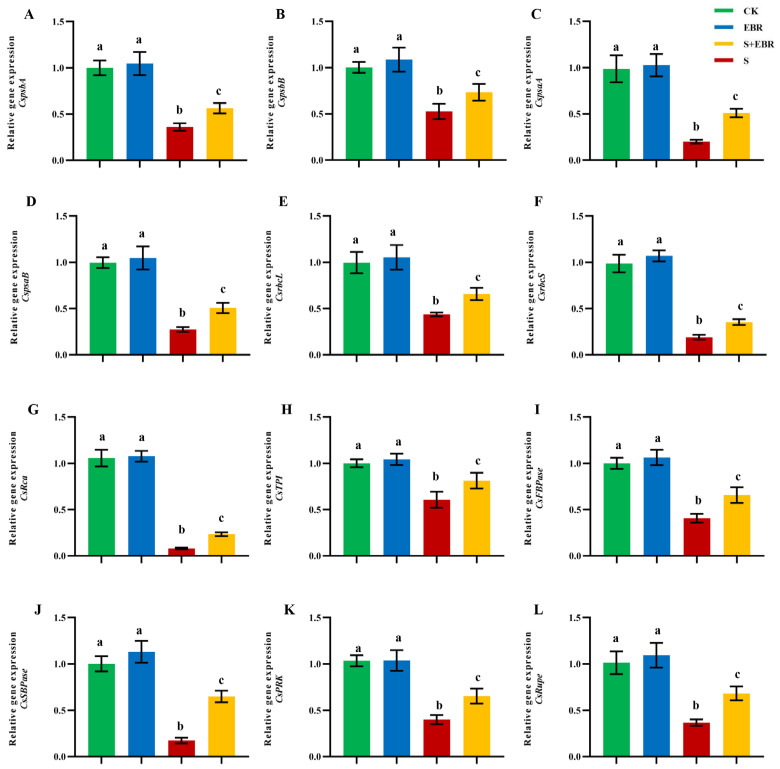
Effects of EBR on the expression of genes related to photosynthesis in cucumber leaves under NaHCO_3_ stress. The relative expression levels of *psbA* (**A**), *psbB* (**B**), *psaA* (**C**), *psaB* (**D**), *rbcL* (**E**), *rbcS* (**F**), *Rca* (**G**), *TPI* (**H**), *FBPase* (**I**), *SBPase* (**J**), *PRK* (**K**), and *Rupe* (**L**) were measured. Abbreviations: *rbcL*, ribulose-1,5-bisphosphate carboxylase/oxygenase large subunit; *rbcS*, ribulose-1,5-bisphosphate carboxylase/oxygenase small subunit; *Rca*, Rubisco activase; *TPI*, triose phosphate isomerase; *FBPase*, fructose-1,6-bisphosphatase; *SBPase*, sedoheptulose-1,7-bisphosphatase; *Rupe*, ribulose-5-phosphate epimerase; *PRK*, phosphoribulokinase. Different letters indicate significant differences (*p* < 0.05; n = 3).

We assessed the expression levels of photosynthesis-related genes in cucumber leaves 48 h post-treatment using quantitative real-time PCR (qRT-PCR), as depicted in Figure 8. Under normal growth conditions, the application of exogenous EBR resulted in a slight enhancement of most photosynthesis-related genes, although these changes were not statistically significant. In contrast, under NaHCO_3_ stress, there was a significant suppression of genes associated with PSII (*psbA* and *psbB*) and photosystem I (PSI) (*psaA* and *psaB*), along with genes involved in the Calvin cycle, such as *rbcL*, *rbcS*, *FBPase*, *SBPase*, *Rupe*, *PRK*, *Rca*, and *TPI*. However, the application of exogenous EBR under these stress conditions significantly counteracted this suppression, markedly enhancing the expression of these crucial photosynthetic genes. This enhancement included notable upregulation of PSII-related genes (*psbA* and *psbB*), PSI-related genes (*psaA* and *psaB*), and Calvin cycle-related genes. Specifically, *rbcL* and *rbcS*, encoding the large and small subunits of ribulose-1,5-bisphosphate carboxylase/oxygenase (Rubisco), respectively, exhibited significant increases. Additionally, substantial enhancements were also observed in the expression levels of *FBPase* (fructose-1,6-bisphosphatase), *SBPase* (sedoheptulose-1,7-bisphosphatase), *Rupe* (ribulose-5-phosphate isomerase), *PRK* (phosphoribulokinase), *TPI* (triose phosphate isomerase), and *Rca*, which encode Rubisco activase, underlining the profound regulatory effects of EBR on photosynthesis under NaHCO_3_-induced stress conditions.

**Figure 9:**
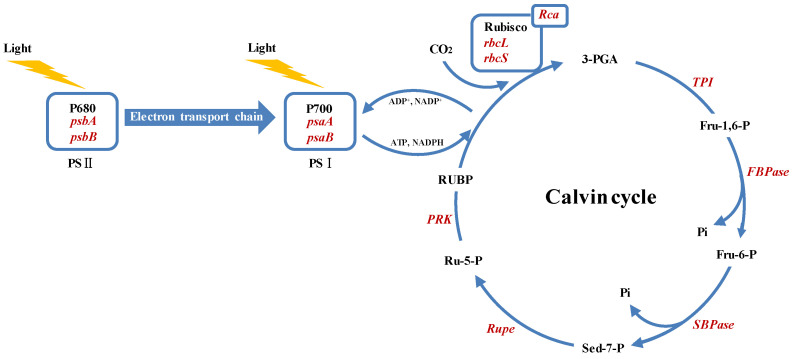
Impact of EBR on photosynthetic electron transport and Calvin cycle in cucumber leaves under NaHCO_3_ stress. Compared to the NaHCO_3_ treatment, the application of exogenous EBR upregulated the expression of crucial photosynthesis-related genes. These include *psbA* and *psbB* associated with PSII; *psaA* and *psaB* integral to photosystem I (PSI); and key genes involved in the Calvin cycle such as *rbcL* and *rbcS*, which encode the large and small subunits of ribulose-1,5-bisphosphate carboxylase/oxygenase (Rubisco), respectively. Additionally, genes like *Rca* (Rubisco activase), *TPI* (triose phosphate isomerase), *FBPase* (fructose-1,6-bisphosphatase), *SBPase* (sedoheptulose-1,7-bisphosphatase), *Rupe* (ribulose-5-phosphate isomerase), and *PRK* (phosphoribulokinase) also showed significant upregulation. The red highlights in the figure indicate areas where the expression levels of these genes were significantly enhanced due to EBR treatment under NaHCO_3_ stress. Key abbreviations: PSII (photosystem II); P680 (primary electron donor of PSII); PSI (photosystem I); P700 (primary electron donor of PSI); rbcL, *rbcS* (genes encoding the large and small subunits of Rubisco, respectively); 3-PGA (3-phosphoglyceric acid); Fru-1,6-P (fructose-1,6-bisphosphate); Fru-6-P (fructose-6-phosphate); Sed-7-P (sedoheptulose-7-phosphate); Ru-5-P (ribulose-5-phosphate); RuBP (ribulose-1,5-bisphosphate); *Rubisco* (ribulose-1,5-bisphosphate carboxylase/oxygenase).

As shown in Figure 9, the application of exogenous EBR significantly alleviated the negative impact of NaHCO_3_-induced alkaline stress on cucumber leaf photosynthesis. Gene expression analysis demonstrated a notable upregulation of key genes associated with PSII and PSI, including *psbA*, *psbB*, *psaA*, and *psaB*, indicating that EBR enhances electron transport efficiency from the primary electron acceptor in PSII to the terminal acceptors at the PSI side (Figure 9). Additionally, EBR treatment significantly increased the expression of essential Calvin cycle genes, such as *rbcL* and *rbcS* (which encode the large and small subunits of Rubisco), as well as other key enzymes including *Rca*, *TPI*, *PRK*, *Rupe*, *FBPase*, and *SBPase*, suggesting an accelerated Calvin cycle and improved carbon fixation efficiency. In contrast, NaHCO_3_ treatment alone resulted in significant suppression of gene expression related to both photosystems and the Calvin cycle, as well as decreased Rubisco activity, leading to reduced electron transport efficiency and lower carbohydrate synthesis. These findings suggest that EBR plays a crucial role in mitigating the inhibitory effects of alkaline stress by enhancing photosynthetic electron transport.

## 3. Discussion

Biomass accumulation serves as an essential indicator for a comprehensive assessment of plant stress tolerance [36]. In our investigation, we found that treatment with exogenous EBR significantly alleviated the growth suppression observed in cucumber seedlings under NaHCO_3_-induced stress. This improvement was attributed to enhanced photosynthetic efficiency and increased reactive oxygen species scavenging capacity, as documented in Figure 1 and Table 1. Our findings are consistent with recent advancements reported in the literature. For instance, Zeng et al. (2024) demonstrated that EBR augments salt stress tolerance in *Quisqualis indica*, a mangrove species, by upregulating S-nitrosoglutathione reductase activity and modulating the nitric oxide signaling pathway [37]. In a similar vein, Chen et al. (2023) observed that EBR application in kiwifruit not only enhanced photosynthetic activity but also reduced hydrogen peroxide concentrations in the foliage, thereby effectively mitigating the detrimental effects of salt stress [38]. In alignment with these findings, both El-Azm and Elhady (2023) and Yu et al. (2023) have confirmed the significant role of EBR in reducing biomass decline due to salt stress in green beans and apple seedlings, respectively [39,40].

The reduction in plant biomass under stress conditions is primarily attributed to the inhibition of photosynthesis. Salt stress induces osmotic stress, leading to a decline in stomatal conductance and transpiration rates, which in turn diminishes photosynthetic activity [41,42]. In salt environments, the imbalance in intracellular Na^+^/K^+^ ratios contributes to stomatal closure, chlorophyll degradation, and photosystem damage [43]. Our findings indicate that NaHCO_3_ stress substantially decreases Pn in cucumber seedlings (Figure 4), primarily due to the inhibition of chlorophyll synthesis and damage to the photosynthetic apparatus, resulting in impaired photosynthetic performance [16,17]. Chlorophyll, the predominant photosynthetic pigment in higher plants, is critical for the absorption, transmission, and distribution of light energy. It transforms light energy into chemical energy, essential for the functionality of the plant’s photosystem and reaction centers [44]. Chlorophyll biosynthesis proceeds through a sequence of enzymatic reactions, delineated as follows: glutamic acid (Glu)→ALA→PBG→UroIII→ProtoIX→Mg-ProtoIX→Pchl→chlorophyll a→chlorophyll b [45]. Environmental and nutritional factors influence the dynamic equilibrium between chlorophyll synthesis and degradation [46,47]. Stress conditions such as salinity often result in reduced chlorophyll content, adversely affecting photosynthesis [48,49]. As shown in Figure 4 and Figure 5, under NaHCO_3_ stress, the decrease in chlorophyll content corresponds with reductions in Pchl, Mg-ProtoIX, UroIII, and ProtoIX, alongside increases in PBG and ALA, indicating that NaHCO_3_ stress blocks the conversion of PBG to UroIII and inhibits its synthesis. The administration of EBR under NaHCO_3_ stress led to increased levels of chlorophyll a, chlorophyll b, Pchl, Mg-ProtoIX, UroIII, and ProtoIX, while levels of PBG and ALA decreased, suggesting that EBR alleviates the inhibition of the chlorophyll synthesis pathway by NaHCO_3_, thereby enhancing chlorophyll content and sustaining normal photosynthesis in cucumber leaves under stress [50,51].

Salt stress causes damage to the plant thylakoid membrane and inhibits enzymes crucial for chlorophyll synthesis, a primary factor behind reduced chlorophyll content under salt stress [50,51]. PBGD and uroporphyrin III synthase (UROS) are pivotal in the conversion of PBG to UroIII [47]. PBGD catalyzes the polymerization of four pheophorbide molecules, followed by UROS catalyzing the formation of cyclic UroIII [52]. Chlorophyll degradation is intricately linked to Chlase activity, typically low due to the spatial separation from chlorophyll [46]. Aging plants or those under adverse stress conditions experience chloroplast structural damage, facilitating chlorophyll contact with chloroplast enzymes and initiating degradation [53]. Salt stress is known to elevate the activity of enzymes associated with chlorophyll degradation [54,55], with post-translational modification of chloroplast enzymes being the rate-limiting step [56]. In our study, NaHCO_3_ stress reduced PBGD activity (Figure 6B), obstructing chlorophyll synthesis at the PBG to UroIII step. Concurrently, NaHCO_3_ stress increased Chlase activity (Figure 6A), hastening chlorophyll degradation, thereby leading to a decrease in chlorophyll content and inhibited photosynthesis (Figure 4). NaHCO_3_ stress not only impedes PBGD activity, resulting in blocked PBG to UroIII conversion, but also exacerbates Na^+^ and ROS toxicity [43], damaging the photosynthetic apparatus and enhancing Chlase activity, thus accelerating chlorophyll degradation. Introduction of exogenous EBR upregulated PBGD activity in cucumber leaves under NaHCO_3_ stress, simultaneously inhibiting Chlase activity. This maintained chlorophyll content stability by promoting synthesis and reducing degradation, thereby protecting the photosynthetic apparatus, enhancing photosynthetic efficiency, and alleviating growth inhibition in cucumbers caused by NaHCO_3_ stress.

Salt and alkaline stresses significantly compromise chloroplast structure, leading to chlorophyll degradation and the inhibition of photosynthesis, ultimately affecting plant growth adversely [57,58]. Our study demonstrates that NaHCO_3_ stress induces a surge in ROS in cucumber plants, as depicted in Figure 2. This stress notably reduces photochemical efficiency (Fv/Fm, ΦPSII, Fv′/Fm′) (Figure 7) and impairs the functionality of the electron transport chain (qP), culminating in oxidative damage to the photosynthetic apparatus [59]. Furthermore, NaHCO_3_ stress disrupts the functional balance between PSI and PSII in cucumber leaves. This is evidenced by the significant reduction in the expression of genes associated with PSII (psbA and psbB) and PSI (psaA and psaB), indicating that the alkaline environment impedes the synthesis of photosynthetic pigments and destabilizes the electron transport chain, further damaging vital photosynthetic structures, including chloroplasts [60].

The application of BRs has been shown to effectively protect the structural integrity of chloroplasts and enhance the efficiency of CO_2_ absorption and utilization by boosting the activities of the cytochrome b6/f complex, D1 and D2 proteins, the large subunit of Rubisco, and carbonic anhydrase, thereby regulating the net photosynthetic rate [28,61]. Additionally, BRs significantly increase the net photosynthetic rate by upregulating the expression of related genes, which in turn improves the photochemical efficiency of PSII, chlorophyll content, and the activity of various key enzymes in the Calvin cycle [61]. The Calvin cycle is a crucial pathway for photosynthetic carbon assimilation and is highly dependent on Rubisco, a key rate-limiting enzyme [62]. The increase in Rubisco and other Calvin cycle enzyme activities is thought to be closely linked to the upregulation of related genes by BRs. These genes include those encoding *rbcL*, *rbcS*, and multiple enzymes associated with the Calvin cycle, such as *TPI*, *FBPase*, and *PRK* [61,63,64]. In our study, as shown in Figure 8, NaHCO_3_ stress downregulated Rubisco-related genes *rbcL*, *rbcS*, and *Rca*, as well as Calvin cycle genes (e.g., *TPI*, *FBPase*, *SBPase*, *Rupe*, and *PRK*), indicating that the carbon fixation process was severely hindered, plant photosynthesis decreased overall, and the carbon assimilation capacity of cucumbers was limited, resulting in inhibited growth [65,66]. These results are consistent with previous findings on the effects of salt stress [67,68]. Under NaHCO_3_ stress, exogenous EBR treatment significantly upregulated the expression of these photosynthesis-related genes, indicating that EBR alleviated the inhibition of the Calvin cycle by NaHCO_3_ stress and enhanced carbon assimilation and photosynthetic capacity. Previous studies have shown that BRs can maintain cellular redox balance by activating the MAPK and NO signaling pathways involved in plant hormone regulation and increasing the activity of antioxidant enzymes, thereby protecting photosynthetic organs from free radical damage and regulating downstream transcription factors that are directly involved in the transcriptional regulation of photosynthetic genes, thus enhancing plant stress resistance [69,70,71]. This EBR-induced hormone regulation and increased antioxidant capacity may represent an important mechanism by which it improves the photosynthetic performance of cucumbers in this study.

Salt and alkaline stress severely inhibits plant photosynthesis and respiration, resulting in impaired electron transport and substantial accumulation of ROS [43]. The primary components of ROS include superoxide anion (O_2_^.−^), hydrogen peroxide (H_2_O_2_), and hydroxyl radicals (OH). While low concentrations of ROS can trigger defense responses and enhance plant resistance, high concentrations may oxidize intracellular macromolecules and plasma membranes, leading to significant cellular damage and structural impairment [72]. ROS accumulation is a critical indicator of plant injury under salt and alkaline conditions [49]. In this study, NaHCO_3_ stress led to a significant increase in ROS levels in cucumber, as illustrated in Figure 2. Exogenous EBR mitigates ROS accumulation induced by abiotic stresses, thereby enhancing plant stress tolerance [19,73]. In this experiment, exogenous EBR upregulated the expression of *SOD* and genes involved in the AsA-GSH cycle (*APX*, *MDHAR*, *DHAR*, *GR*) (Figure 3), enhancing antioxidant capacity and maintaining the stability of the AsA-GSH cycle, thus effectively reducing the ROS levels under NaHCO_3_ stress and mitigating oxidative damage. Similar effects of exogenous BR have been observed in drought-treated barley [74] and salt-stressed perennial ryegrass [75].

Exogenous BR not only alleviates the inhibition of photosynthesis in cucumber seedlings under NaHCO_3_ stress but also enhances their capacity to scavenge ROS, thereby reducing the growth inhibition experienced by cucumber seedlings under these stress conditions.

## 4. Materials and Methods

### 4.1. Experimental Materials and Design

This study was conducted in greenhouses at Shandong Agricultural University and the Shandong Institute of Sericulture using the cucumber cultivar Jinyan No. 4. Seeds were pre-soaked in warm water at 28 °C for 4 h, disinfected with a 0.3% sodium hypochlorite solution for 30 s, and thoroughly rinsed. After disinfection, the seeds were placed between two moistened layers of filter paper and incubated in darkness at 28 °C for germination. Once the cotyledons had fully expanded, uniformly germinated seedlings were selected, carefully washed to remove residual root substrate, and transplanted into hydroponic pots containing 5 L of Hoagland nutrient solution, with 4 seedlings per pot.

Experimental treatments began 8 days after pre-treatment and included the following groups:

CK: control plants grown in complete Hoagland nutrient solution;

EBR: Hoagland nutrient solution supplemented with 0.2 μmol/L EBR;

S: Hoagland nutrient solution supplemented with 30 mmol/L NaHCO_3_;

S + EBR: Hoagland nutrient solution supplemented with 30 mmol/L NaHCO_3_ and 0.2 μmol/L exogenous EBR.

The experiment followed a randomized block design with three replicates per treatment. Each replicate consisted of 15 pots, resulting in a total of 180 plants per treatment group (4 plants per pot × 15 pots × 3 replicates). EBR was applied daily, and nutrient solutions (including NaHCO_3_, where applicable) were replaced every 2 days, with intermittent aeration provided. Sampling for photosynthetic parameters, chlorophyll fluorescence characteristics, and chlorophyll and chlorophyll precursor content was conducted on days 0, 3, 6, 9, 12, and 15 of the treatment period. Chlase and PBGD activities were measured on days 7 and 15. On day 10 of treatment, ROS (H_2_O_2_ and O_2_^.−^) staining was performed. On day 15 of treatment, plant phenotypic images were captured, and biomass was determined.

### 4.2. Plant Biomass Measurement

Fifteen days after treatment, the plants were rinsed with deionized water, blotted dry with absorbent paper, and their biomass was assessed following the separation of aboveground and belowground components.

### 4.3. Photosynthetic Parameters Measurement

Photosynthetic rate (Pn), transpiration rate (Tr), and stomatal conductance (Gs) were measured using a Li-6400 photosynthesis system (Li-COR Biosciences, Lincoln, NE, USA) on the second functional leaf below the growing point.

### 4.4. Chlorophyll Content Determination

To determine the chlorophyll content, weigh 0.5 g of cucumber leaves, and cut them into small pieces in a mortar. Add a small amount of fine quartz sand, and grind the mixture into a paste. Extract the chlorophyll gradually with an 80% acetone aqueous solution until the residue becomes colorless. Filter the extract, and dilute it to a final volume of 15 mL. Measure the absorbance at 663 nm and 645 nm using the GENESYS 150 UV-Visible Spectrophotometer (Thermo Fisher Scientific, Waltham, MA, USA) and the UV-751 spectrophotometer.. The resulting optical density (OD) values are used in the following formulas to calculate the concentrations of chlorophyll a and chlorophyll b, as well as the chlorophyll a + b:

chlorophyll a content = 12.7 × OD663 − 2.69 × OD645

chlorophyll b content = 22.9 × OD645 − 4.86 × OD663

chlorophyll a + b content = 20.2 × OD663 + 8.02 × OD645

### 4.5. Chlorophyll Fluorescence Parameter Measurement

Chlorophyll fluorescence parameters Fo, Fm, Fs, Fo′, and Fm′ were assessed using an FMS-2 portable modulation fluorometer (FluorCam 7; Photon Systems Instruments, Brno, Czech Republic). Calculations were based on methods from Gong (2014) [17] and others, including maximum photochemical efficiency Fv/Fm = (Fm − Fo)/Fm; actual photochemical efficiency ΦPSII = (Fm′ − Fs)/Fm′; antenna conversion efficiency Fv′/Fm′ = (Fm′ − Fo′)/Fm′; and photochemical quenching coefficient qP = (Fm′ − Fs)/(Fm′ − Fo′).

### 4.6. Determination of the Content of Chlorophyll Precursor Substances

The determination of ALA was performed according to the method of Morton [76], with slight modifications. Specifically, 2 g of fresh sample was added to 6 mL of sodium acetate buffer (pH 4.6) and thoroughly ground while kept in an ice bath. After boiling the mixture in a water bath for 15 min, it was centrifuged at 10,000× *g* for 20 min. The precipitate was washed twice with 4 mL of extraction solution, and the supernatant was combined. Subsequently, 1 mL of the extract was taken, and 4 drops of ethyl acetoacetate were added. The mixture was then boiled in a water bath for 15 min before adding an equal volume of Alder’s reagent. The absorbance was measured at 553 nm after 15 min. A standard curve was prepared using ALA-HCl (Sigma-Aldrich, St. Louis, MO, USA) as a standard sample to calculate the ALA content (nmol/g FW).

The content of UroIII and PBG was determined by following the method of Bogorad (1962) [77]. For the determination of UroIII, 1 g of fresh sample was weighed, and 5 mL of Tris-HCl (pH 7.2) buffer was added. The mixture was ground and extracted in an ice bath. After centrifugation at 5000× *g* for 15 min at 4 °C, the supernatant was taken, and 1 drop of glacial acetic acid was added to adjust the pH to 4.0. The mixture was centrifuged again at 5000× *g* for 15 min at 4 °C, and the supernatant was discarded. The precipitate was washed twice with 5 mL of water and centrifuged again to obtain a uracil-based precipitate. To extract uracil, 4 mL of pre-cooled concentrated ammonia water was added twice, and the supernatant was combined and transferred to an evaporating dish. The solution was evaporated to dryness at 55 °C, followed by the addition of 4 mL of 5% methanol sulfamate and incubation for 48 h. Afterward, 20 mL of water was added, and the pH was adjusted to 4.0 with saturated sodium acetate. The mixture was extracted twice with 4 mL of chloroform, evaporated to dryness at 55 °C, dissolved in 4 mL of chloroform, centrifuged at 5000× *g* for 10 min, and the absorbance was measured at 405.5 nm. Each treatment was repeated three times, and the content (nmol/g FW) was calculated using the following formula:

UroIII = 5.48A405.5 × 10 × 0.004 × 3 × 1000/FW

For the determination of PBG, 1 g of fresh sample was weighed, and 5 mL of Tris-HCl (pH 8.2) buffer was added, followed by grinding and extraction in an ice bath. The mixture was then centrifuged at 12,000× *g* and 4 °C for 10 min. The supernatant was taken, and 1 drop of glacial acetic acid was added to adjust the pH to 4.0 before centrifugation again. After discarding the supernatant, the precipitate was washed to obtain a PBG precipitate. To extract PBG, 4 mL of pre-cooled concentrated ammonia water was added twice, and the supernatant was combined and evaporated to dryness. The residue was treated with 4 mL of 5% sulfuric acid–methanol for esterification for 48 h. After adding 20 mL of water and adjusting the pH, the mixture was extracted with 4 mL of chloroform, and the colorimetric absorbance was finally measured at a wavelength of 553 nm, each treatment was repeated three times, and the PBG content was calculated using the following formula (nmol/g FW):

PBG = A553 × 1.383 × 10 × 0.004 × 3 × 1000/FW

ProtoIX, Mg-ProtoIX, and Pchl were determined using the method of Hodgins and VanHuystee (1986) [45]. A fresh leaf sample without main veins weighing 0.5 g was taken, and 80% alkaline acetone (volume ratio 80:20 of acetone to 1% ammonia solution) was added. After grinding and extracting in an ice bath, the mixture was centrifuged at 15,000× *g* for 15 min at 4 °C. The supernatant was transferred to a 10 mL volumetric flask, made up to the mark with 80% alkaline acetone, shaken well, and the absorbance values were measured at wavelengths of 575 nm, 590 nm, and 628 nm.

The following equations were used to calculate the contents of UroIII, PBG, ProtoIX, Mg-ProtoIX, and Pchl, with three replicates for each treatment. Here, ‘A’ represents the absorbance measured at the specified wavelength (e.g., 405.5 nm, 553 nm, etc.); ‘V’ is the final volume (mL) of the extracted solution; and ‘FW’ is the fresh weight of the sample (g).

Mg-ProtoIX = (0.06077 × A590 − 0.01937 × A575 − 0.003423 × A628) × V/FW

ProtoIX = (0.18016 × A575 − 0.04036 × A628 − 0.04515 × A590) × V/FW

Pchl = (0.03563 × A628 + 0.007225 × A590 − 0.02955 × A575) × V/FW

### 4.7. Chlorase and Porphobilinogen Deaminase Activity

Chlase activity was determined using the method established by Holden (1961) [78] and further adapted by Zhou et al. (2016) [79]. A sample of 0.5 g of fresh cucumber leaves was weighed and ground in 20 mL of 50% ethanol. This mixture was then combined with 8 mL of a chlorophyll solution and 4 mL of a sodium citrate solution (0.04 M), and the pH was adjusted to approximately 8. The sample was incubated in the dark for 18 h. Following incubation, the mixture was filtered through a funnel to remove pigments. The filtrate was subsequently mixed with 10 mL of 2% sodium chloride and diluted to a final volume of 50 mL with 80% ethanol. The decomposition activity was assessed by measuring the absorbance at 607 nm using a spectrophotometer. Enzyme activity was expressed in µmol chlorophyllamide mg^−1^ FW min^−1^, with one unit defined as the amount of enzyme that catalyzes the decomposition of 10% of the substrate to chlorophyllamide within one hour.

PBGD activity was quantified following the protocols of Rimington (1960) [80], Frydman and Frydman (1979) [81], and Zhou et al. (2016) [79]. A sample of 4.0 g of cucumber leaves was extracted using 20 mL of phosphate buffer (pH 6.8) to obtain the substrate (PBG). The material was ground in a mortar and pestle, and the supernatant was collected after centrifugation at 12,000× *g* for 10 min for subsequent analysis. Additionally, 1.0 g of cucumber material was ground in a mortar and pestle, and 10 mM Tris-HCl (pH 7.4, 4 °C) was added. This mixture was then centrifuged at 20,000× *g* for 15 min, and the supernatant was retained. In a test tube, 1 mL of PBG was combined with 1 mL of PBGD and 2 mL of phosphate buffer (pH 7.4). The reaction was conducted at 4 °C for 60 min. After adding Ehrlich’s reagent (Frydman and Frydman, 1979), the absorbance was measured at 553 nm. Enzyme activity was expressed in nmol g^−1^ protein h^−1^ based on the absorbance changes induced by the reaction.

### 4.8. O_2_^.−^ and H_2_O_2_ Staining

ROS staining, specifically for O_2_^.−^ and H_2_O_2_, was conducted using an improved method based on Xia et al. (2009) [28].

O_2_^−^ was detected in situ utilizing the NBT staining method. Leaf discs were vacuum-impregnated with 0.1 mg/mL NBT in 25 mM potassium phosphate buffer (pH 7.8) and incubated at room temperature in the dark for 4 h. Following staining, the leaf discs were washed with 80% ethanol at 70 °C for 10 min and subsequently mounted for photography.

H_2_O_2_ was detected in situ using the DAB staining method. Leaf discs were immersed in a 1 mg/mL DAB solution in 50 mM Tris-acetate buffer (pH 3.8) under vacuum and incubated at room temperature in the dark for 24 h. After incubation, the leaf discs were removed and washed with 80% ethanol, then observed under a microscope.

### 4.9. Fluorescence Quantification and Gene Expression Analysis Based on qRT-PCR

Total RNA was extracted from the plant samples using the TRIzol method, and complementary DNA (cDNA) was synthesized with the RevertAid First Strand cDNA Synthesis Kit (Thermo Fisher Scientific, Waltham, MA, USA). Quantitative real-time polymerase chain reaction (qRT-PCR) analysis was performed using the Power SYBR Green PCR Master Mix (Applied Biosystems, Foster City, CA, USA). The primers used in this study are listed in Table 2.

### 4.10. Data Processing

In this study, a completely randomized experimental design was employed, with each treatment group consisting of three replicates. The data were expressed as mean values with the corresponding standard error. To identify significant differences among the means, Duncan’s New Multiple Range Test was conducted using SPSS software version 22.0. Statistical significance was established with a *p*-value of less than 0.05. Furthermore, for additional data analysis and chart generation, Excel 2010 was utilized.

## 5. Conclusions

This study demonstrates that the exogenous application of EBR effectively mitigates the detrimental effects of NaHCO_3_-induced alkaline stress in cucumber seedlings. EBR contributes to the restoration of growth and photosynthetic efficiency in stressed plants. Notably, it significantly enhances chlorophyll content, net photosynthetic rate (Pn), stomatal conductance (Gs), and crucial indicators of chlorophyll fluorescence, including maximum photochemical efficiency (Fv/Fm) and actual photochemical efficiency (ΦPSII). Additionally, EBR diminishes the accumulation of ROS and favorably regulates the expression of genes involved in the antioxidant ascorbate–glutathione (AsA-GSH) cycle, thereby mitigating oxidative stress. Moreover, EBR administration reverses the inhibition of chlorophyll biosynthesis pathways, primarily through elevated activity of PBGD and reduced chlorophyllase activity. This alteration leads to increased levels of chlorophyll precursors such as UroIII and ProtoIX, thereby enhancing chlorophyll accumulation under alkaline conditions.

Crucially, EBR stabilizes the photosynthetic electron transport system and enhances the expression of essential photosynthesis-related genes, improving light energy utilization and accelerating the Calvin cycle. Collectively, these findings underscore the potential of brassinosteroids to bolster plant resilience against harsh environmental conditions by reinforcing photosynthetic and antioxidant systems, thereby offering substantial agricultural benefits under alkaline stress.

## Figures and Tables

**Table 1:** Effects of exogenous EBR on the growth of cucumber seedlings under NaHCO_3_ stress.

Treatment	Plant Height (cm)	Shoot Fresh Weight (g·Plant^−1^)	Root Fresh Weight (g·Plant^−1^)	Total Fresh Weight (g·Plant^−1^)
CK	50.21 ± 0.73 a	32.74 ± 2.56 b	13.34 ± 1.03 a	46.08 ± 2.81 a
EBR	51.40 ± 0.67 a	34.26 ± 2.92 a	13.61 ± 1.80 a	47.87 ± 4.71 a
S	25.27 ± 4.12 c	11.67 ± 1.99 d	3.78 ± 0.43 c	15.45 ± 2.26 c
S + EBR	39.65 ± 4.68 b	15.51 ± 1.93 c	6.03 ± 0.40 b	21.54 ± 2.32 b

Note: different lowercase letters in the same column indicate significant differences at the 0.05 level among treatments. (CK denotes control plants grown under normal Hoagland nutrient solution; EBR denotes the addition of EBR under normal growth conditions; S denotes NaHCO_3_ stress treatment; S + EBR denotes the addition of EBR under NaHCO_3_ stress).

**Table 2:** Primer sequences.

Gene Name	Primer Sequences
*actin*	F: ATGGCCGATGCCGAGGATATR R: TAGGAGCATCATCACCAGCAAAAC
*CsCu-ZnSOD*	F: CTCCATTTTCAATCTCTCATTATCC R: ATAGAAGTGATTGTGCGGCCATAG
*CsFe-SOD*	F: TACACTGAACCTGAGTTCAACAAC R: ACGTTCTCTGAGAAACCAAACAGA
*CsAPX*	F: CTGCTACTGTTTTTGGAACCGCCG R: GCGGAGGAGAGGAAACGAGTAGTT
*CsMDHAR*	F: ACAGCCTTCTTCTGTTGCCTTCAG R: CTCTATTGTCGTTGGCGAAATCCG
*CsDHAR*	F: ATGTCGGGCTCCAGAATCCAACCA
	R: AAAGCGAGGAATTGGAAGGAAGGT
*CsGR*	F: GTCCGATAGTGCTGGAGGTGTTGG
	R: CCATCCAAAGCCATGACTCTCTTC
*CspsbA*	F: GTATTCCAGGCTGAGCACAACATC
	R: TACCTAAAGCGGTGGACCAGATAC
*CspsbB*	F: GGTATTTGGAGTTACGAAGGTGTG
	R: CCCAACCCTGAGAGAAATAAATGA
*CspsaA*	F: GATTTCTCATAGTTGGTGCTGCTG
	R: TACAAACCAAAACTGTGAAAGCCT
*CspsaB*	F: ATTTGGACATCTTGTTTGGGCTAC
	R: TGATGTAGAGGCAATCAAGAAAGC
*CsrbcL*	F: TACTGATATCTTGGCAGCATTCCG
	R: AAGATTCAGCAGCTACAGCGGC
*CsrbcS*	F: ATGGCTTCATCCATTCTCTCATCC
	R: CCAGTGAATGGTGCTACCATGCTA
*CsRca*	F: GAATATGGCAACATGCTCGTCATG
	R: TCCAAGAGCAGCTTCGTTCAGAT
*CsTPI*	F: CTCTCTTTCACAACGTCCACTCACA
	R: ACCAACGAAGAACTTGCCGGAG
*CsFBPase*	F: AATCTCTCGTTCTCTCTCTCGCCTC
	R: CGCTATGCCTTCTATTTCCACGG
*CsSBPase*	F: CAGTGTCCTCCTCATACTTGGGTTG
	R: CTGGGAAGAAAGATTGGGGAGAAA
*CsRupe*	F: TCCCAAGTCAGTGGGTTTATCGGAG
	R: AACCTTCTCCTCGAAACGGTAAGAG
*CsPRK*	F: ATCCACACCCTCATTCATTTCTCC
	R: GCAGTTGAGGGAGTGAAGAAGAAGA

## Data Availability

The study’s data can be obtained by contacting the corresponding author upon request.
